# GSE Is a Maternal Factor Involved in Active DNA Demethylation in Zygotes

**DOI:** 10.1371/journal.pone.0060205

**Published:** 2013-04-01

**Authors:** Yuki Hatanaka, Natsumi Shimizu, Satoshi Nishikawa, Mikiko Tokoro, Seung-Wook Shin, Takuji Nishihara, Tomoko Amano, Masayuki Anzai, Hiromi Kato, Tasuku Mitani, Yoshihiko Hosoi, Satoshi Kishigami, Kazuya Matsumoto

**Affiliations:** 1 Division of Biological Science, Kinki University Graduate School of Biology-Oriented Science and Technology, Kinokawa, Wakayama, Japan; 2 Institute of Advanced Technology, Kinki University, Kainan, Wakayama, Japan; The Babraham Institute, United Kingdom

## Abstract

After fertilization, the sperm and oocyte genomes undergo extensive epigenetic reprogramming to form a totipotent zygote. The dynamic epigenetic changes during early embryo development primarily involve DNA methylation and demethylation. We have previously identified *Gse* (gonad-specific expression gene) to be expressed specifically in germ cells and early embryos. Its encoded protein GSE is predominantly localized in the nuclei of cells from the zygote to blastocyst stages, suggesting possible roles in the epigenetic changes occurring during early embryo development. Here, we report the involvement of GSE in epigenetic reprogramming of the paternal genome during mouse zygote development. Preferential binding of GSE to the paternal chromatin was observed from pronuclear stage 2 (PN2) onward. A knockdown of GSE by antisense RNA in oocytes produced no apparent effect on the first and second cell cycles in preimplantation embryos, but caused a significant reduction in the loss of 5-methylcytosine (5****mC) and the accumulation of 5-hydroxymethylcytosine (5****hmC) in the paternal pronucleus. Furthermore, DNA methylation levels in CpG sites of LINE1 transposable elements, *Lemd1*, *Nanog* and the upstream regulatory region of the *Oct4* (also known as *Pou5f1*) gene were clearly increased in GSE-knockdown zygotes at mid-pronuclear stages (PN3-4), but the imprinted H19-differential methylated region was not affected. Importantly, DNA immunoprecipitation of 5****mC and 5****hmC also indicates that knockdown of GSE in zygotes resulted in a significant reduction of the conversion of 5****mC to 5****hmC on LINE1. Therefore, our results suggest an important role of maternal GSE for mediating active DNA demethylation in the zygote.

## Introduction

Resetting and modification of epigenetic marks as a part of epigenetic reprogramming occur in the genomes of early embryos and primordial germ cells (PGCs) [1]–[5]. The genome-wide reprogramming of DNA methylation/demethylation and histone modification works in concert to establish each cell lineage during embryogenesis. Unraveling the mechanism of epigenetic reprogramming will provide important information on the characterization of germ cells, comprising totipotent and unipotent cells possessing an underlying genomic plasticity [6], [7].

Both sperm and oocyte genomes undergo epigenetic reprogramming under the control of maternal factors immediately after fertilization. The rapid reduction of 5-methylcytosine (5****mC) content in the paternal genome before first DNA replication is the reset of epigenetic memory, classified as active DNA demethylation [1], [3], [8]. This DNA demethylation of the paternal genome is linked to global conversion of 5****mC to 5-hydroxymethylcytosine (5****hmC) catalyzed by the maternally encoded enzyme Tet methylcytosine dioxygenase 3 (Tet3), which is intensely expressed in oocytes and zygotes [9]–[11]. By contrast, the maternal genome is protected from the fast genome-wide DNA demethylation at the zygote stage and is slowly demethylated in a passive (replication-dependent) manner [12]. Maternal factor PGC7/Dppa3/Stella protein is also required for protecting the conversion from 5****mC to 5****hmC by strongly associating with maternal chromatin regions marked with dimethylated histone H3 lysine 9 (H3K9me2) [10], [11]. Interestingly, the PGC7/Dppa3/Stella protein is suggested to inhibit recruitment of Tet3 to the chromatin of the maternal pronucleus by altering chromatin configuration [12], [13]. However, the mechanisms underlying DNA demethylation in early embryogenesis are not well understood [14].

We have previously identified *Gse* (gonad-specific expression gene) as expressed specifically in the germ cells of adult ovaries and testes, although it has not yet been characterized functionally [15]. The GSE protein shows a distinctive subcellular localization pattern in oocytes and early embryos. After fertilization, GSE, which diffuses into the cytoplasm of MII oocytes, clearly accumulates in both pronuclei. A predominant localization of GSE in the nucleus is observed throughout the subsequent cleavage stages. At the blastocyst stage, a strong signal of GSE is observed in the nuclei of the inner cell mass cells, whereas only a very weak signal is detected in the trophectoderm cells. This appears to correlate with the site of action of GSE and led us to investigate the possible role of GSE in DNA demethylation in early mouse embryos.

In this study, we identified a strong association of GSE with nucleosome architecture in the paternal pronucleus of the zygote and related this to a dramatic loss of 5****mC and an accompanying conversion of 5****mC to 5****hmC. We also confirmed the presence of GSE-mediated DNA demethylation by bisulphite sequencing and the conversion of 5****mC to 5****hmC by methylated and hydroxymethylated DNA immunoprecipitation (MeDIP and hMeDIP). These results suggest an important function of GSE in active DNA demethylation during zygote development.

## Materials and Methods

### Animals

All mice (ICR strain) were purchased from Kiwa Experimental Animals (Wakayama, Japan) and maintained in light-controlled, air-conditioned rooms. This study was carried out in strict accordance with the recommendations in the Guidelines of Kinki University for the Care and Use of Laboratory Animals. The protocol was approved by the Committee on the Ethics of Animal experiments of Kinki University (Permit Number: KABT-19-003). All mice were killed by cervical dislocation, and all efforts were made to minimize suffering and to reduce the number of animals used in the present study.

### Collection of Oocytes, in vitro Fertilization and Embryo Culture

Collection of spermatozoa, oocytes and fertilized embryos was performed as described [15]. In brief, spermatozoa were collected from the cauda epididymidis of male mice. The sperm suspension was incubated in HTF medium for 1.5 h to allow for capacitation at 37°C under 5% CO_2_ in air. Oocytes were collected from the excised oviducts of female mice (2–3 months old) that had been superovulated with pregnant mare serum gonadotropin (PMSG; Serotropin, Teikoku Zoki, Tokyo, Japan) followed 48 h later with human chorionic gonadotropin (hCG; Puberogen, Sankyo, Tokyo, Japan). Cumulus-oocyte complexes were recovered into pre-equilibrated HTF medium. The sperm suspension was added to the oocyte cultures, and morphologically normal fertilized oocytes were collected 2 h after insemination. The fertilized embryos were cultured in KSOM medium [16] at 37°C under 5% CO_2_ in air.

### Western Blot Analysis

The procedures were essentially performed as described [15], [17]–[19]. In brief, the samples (30 cells of each embryonic stage) were subjected to sodium dodecyl sulfate (SDS) polyacrylamide gel electrophoresis. The proteins were resolved in 15% running gels and electrophoretically transferred to polyvinylidene difluoride (PVDF) membranes (GE Healthcare, Little Chalfont, UK). The membranes were incubated in Block Ace (Dainippon-Pharm, Osaka, Japan) at room temperature (RT) for 1 h. They were washed with phosphate-buffered saline containing 0.2% Tween 20 (PBST) and incubated at 4°C overnight with anti-GSE antiserum (1∶2,000), and with anti-actin antibody (Santa Cruz Biotechnology, Heidelberg, Germany; sc-1616) as a loading control. The membranes were washed in PBST, incubated with donkey anti-rabbit IgG-horseradish peroxidase (HRP) conjugate (1∶50,000; Millipore Corp., Billerica, MA, USA; AP182P) and donkey anti-goat IgG HRP conjugate (1∶50,000; Millipore Corp.; AP180P) at RT for 1 h, washed with PBST and developed using ECL Prime Western Blotting detection reagent (GE Healthcare).

### Immunocytochemistry and Microscopy

The classification of pronuclear stages (PN) stages was performed according to previous study [8], [11], where the pronuclear morphology and hours post-insemination was taken into consideration. Subcellular localizations of GSE, H3, 5****mC and 5****hmC were determined by immunocytochemical analysis of oocytes and early embryos, as described [20]. Embryos were fixed in 4% paraformaldehyde (PFA; Nacalai Tesque, Kyoto, Japan) in phosphate-buffered saline (PBS) at RT for 10 min, and the fixed samples were then incubated in PBS containing 0.1–0.2% Triton X-100 (Nacalai Tesque) at RT for 1 h. For 5****mC and 5****hmC, the specimens were incubated in 4 N HCl at RT for 30 min and then incubated in 0.1 M EDTA at RT for 30 min. They were then incubated with anti-GSE antiserum (final dilution, 1∶2000), anti-H3 antibody (final dilution, 1∶1000; MAB, Sapporo, Hokkaido, Japan; MA301B), anti-5****mC antibody (final dilution, 1∶2000; Calbiochem, Darmstadt, Germany; NA81) or anti-5****hmC antibody (final dilution, 1∶2000; Active Motif, Carlsbad, CA, USA; 39769) in PBS containing 30 mg/ml bovine serum albumin at 4°C overnight (Table S1). After incubation, the cells were reacted with an Alexa Fluor 594-labeled donkey anti-rabbit IgG secondary antibody for anti-GSE and 5****hmC (final dilution, 1∶4000; Invitrogen, Carlsbad, CA, USA; A-21207), with an Alexa Fluor 488-labeled donkey anti-mouse IgG secondary antibody (final dilution, 1∶4000; Invitrogen; A21202) for anti-H3, and with an Alexa Fluor 350-labeled goat anti-mouse IgG secondary antibody (final dilution, 1∶4000; Invitrogen; A-11045) for 5****mC, all at RT for 1 h. Specimens were mounted on glass slides in Vectashield mounting medium (Vector Laboratories, Burlingame, CA, USA) containing 2–5 µg/ml DAPI (Invitrogen; D1306) (Table S1). Finally, the slides were imaged using an Olympus BX51 microscope (OLYMPUS, Tokyo, Japan) equipped with an Olympus DP 70 digital camera (OLYMPUS). ImageJ software (NIH, Bethesda, MD, USA; http://rsbweb.nih.gov/ij/) was used to quantify DAPI staining and antibody signals in the center section of each pronucleus. At least two or three independent experiments were performed.

### Triton Treatment of Zygotes before PFA Fixation

Triton treatment of zygotes was performed as described [13]. Zygotes were treated with 0.2% Triton X-100 in PBS for 30 s. They were then washed with PBS three times and then fixed in 4% PFA. After washing with PBS, immunostaining was performed as described above.

### Coimmunoprecipitation

Coimmunoprecipitation was performed as described [21]. We used antibodies against GSE, H3 (1 µg; MAB; MA301B) and H4 (2 µg, Santa Cruz Biotechnology Inc., Santa Cruz, CA, USA; sc-10810). For immunoblot analysis, we used a donkey anti-rabbit IgG–HRP conjugate (1∶50,000; Millipore Corp.; AP182P) for anti-GSE and anti-H4, and a donkey anti-mouse IgG HRP conjugate (1∶50,000; Millipore Corp.; AP192P) for anti-H3, as secondary antibodies (Table S1).

### Recombinant Protein Expression and Purification

GSE cDNA was cloned into the pIRES2-EGFP (Clontech, Palo Alto, CA, USA) vector containing an N-terminal His tag. The His-tagged GSE expression vector was transfected into HEK293 cells using Lipofectamine 2000 Reagent (Invitrogen). The protein solution was extracted from the HEK293 cell with 0.3% NP40 containing 150 mM NaCl and 20 mM Tris-HCl. The recombinant protein was purified with a 100 mM phosphate buffer containing 500 mM NaCl using TALON Metal Affinity Resin (Clontech) and eluted by His TALON Gravity Column (Clontech).

### In vitro Binding Assay of Recombinant His-tagged GSE to H3 or H4 Peptides

N-terminal (residues 1–100) of human H3 or H4 peptides (Abcam, Cambridge, UK) and His-tagged GSE protein were incubated with Dynabeads Protein G (Invitrogen) in a binding buffer containing 0.3% NP40, 150 mM NaCl and 20 mM Tris-HCl at 4°C for 1 h with rotation. Coimmunoprecipitation was performed as described above. Three independent experiments were performed.

### In vitro mRNA Synthesis

GSE antisense RNA and enhanced green fluorescence protein (EGFP) mRNA amplification was performed using Ampliscribe T7 Transcription kits (Epicentre Technologies, Madison, WI, USA) from pcDNA3.1/antisense GSE and pcDNA3.1/EGFP vectors. For efficient translation of the proteins in oocytes or embryos, the 5′ end of each RNA was capped using RNA Cap Analog kits (Epicentre Technologies), according to the manufacturer’s protocol.

### Comicroinjection of GSE Antisense RNA and EGFP mRNA

M II oocytes coinjected with GSE antisense RNA and EGFP RNA for knockdown were cultured in KSOM medium, selected by detection of EGFP fluorescence and then cultured until use, as described [17], [22]. To determine the knockdown efficiency, proteins isolated from 30 EGFP-positive oocytes were used for immunoblotting. The EGFP-positive oocytes were also used for in vitro fertilization (IVF) after laser perforation of the zona pellucida as shown as below.

### Laser Perforation of the Zona Pellucida

The MII oocytes injected with the RNAs (above) were perforated using laser equipment (XYClone, Nikko Hansen Co., Ltd, Osaka, Japan). Laser perforation was performed as described previously [23]. In brief, the laser was applied to the point on the zona pellucida that showed the widest perivitelline space, and a hole (6 µm) was perforated in each (wave length 1,480 nm; output 300 mW; pulse width 120 µs).

### Densitometric Quantification Analysis

Densitometric quantification analysis of the immunoblot bands was performed using a Molecular Imager FX with Quantity One software (BioRad, Hercules, CA, USA).

### Quantitative Real-time RT-PCR Analyses

Quantitative RT-PCR analyses were performed as described [24]. Total RNA was isolated from 30 pooled oocytes or embryos by using RNAqueous micro kits (Ambion, Austin, TX, USA). In brief, cDNA was synthesized from total RNA using High Capacity cDNA Reverse Transcription kits (Applied Biosystems, Foster City, CA, USA). Prepared cDNA samples were amplified and analyzed by quantitative RT-PCR. The primers used are described in Table S2. Amplifications were run in a 7300 ABI Prism Sequence Detector (Applied Biosystems).

### Bisulphite Genomic Sequencing

Genomic DNA from 60 pooled embryos was extracted by using EZ DNA Methylation-Direct kits (Zymo Research Corp., Irvine, CA, USA) according to the manufacturer’s instructions and our previous report [25]. In brief, the samples made up to 20 µl were treated with 2× M-digestion buffer and proteinase K at 50°C for 20 min. The reaction samples were incubated with 130 µl of CT conversion reagent solution at 98°C for 8 min and at 64°C for 3.5 h. After incubation, bisulphite-modified samples were transferred directly to Zymo-Spin IC columns pre-loaded with 600 µl M-binding buffer and inverted several times. Columns were centrifuged at 10,000×g for 30 s, the flow-through was discarded and the column was washed with 100 µl M-wash buffer and centrifuged at 10,000×g for 30 s; 200 µl M-desulphonation buffer was added to the column and allowed to stand at RT for 25 min. The column was washed with 200 µl M-wash buffer and centrifuged at 10,000×g for 30 s twice. Genomic DNA was eluted with 10 µl M-elution buffer by centrifuging at 10,000×g for 30 min. PCR reactions were performed using TaKaRa Taq Polymerase Hot Start Version DNA (TaKaRa Bio Inc., Shiga, Japan). The specific primers are shown in Table S2 and were based on previous reports [9], [26]. Sequence analysis of the PCR product was performed after ligating the amplicon into the plasmid vector, pGEM-T Easy (Promega), and cloning. Three independent experiments were performed.

### DNA Immunoprecipitation with Anti-5****mC and Anti-5****hmC Antibodies

The procedures were essentially performed according to a previous report [9]. In brief, in order to extract the DNA, samples were incubated in a proteinase K (Roche, CH-4070, Basel, Switzerland) solution including 1×SSC, proteinase K (50 ng) and 10% SDS for 1 h at 37°C. Each sample was incubated with RNase for 1 h at 37°C to remove total RNA. The samples were purified by phenol-chloroform treatment and ethanol precipitation with glycogen. The samples were fragmented by AluI digestion for overnight at 37°C and purified by phenol-chloroform treatment and ethanol precipitation with glycogen. After being heat-denatured for 10 min at 95°C, the immunoprecipitation of prepared DNA samples was performed as described above using 1µg of anti-5****mC or anti-5****hmC antibodies and 10 µl Dynabeads. Quantitative RT-PCR analyses were performed as described above. The primers used in this study are described in Table S2. Three independent experiments were performed.

### Statistical Analysis

For statistical analysis, we used StatView version 5.0 (SAS Institute, Cary, NC, USA) and Microsoft Excel and performed analysis of variance (ANOVA) with an α level of 0.05 to determine possible statistically significant differences between group means.

## Results

We have shown previously that mouse GSE is expressed specifically in germ cells and early embryos [15]. Using the Basic Local Alignment Search Tool (BLAST), GSE proteins in different species were searched from the GenBank database (http://www.ncbi.nlm.nih.gov). Alignment of the mouse GSE protein with rat, human, pig and bovine GSE proteins showed 86, 70, 68 and 60% identity at the amino acid level over the entire polypeptide, respectively (Figure S1A). The mouse GSE protein had clear orthologs in other vertebrates such as *Xenopus* (38% identity) and *zebrafish* (30% identity) and the ascidian *Ciona intestinalis* (30% identity) (Figure S1B). Higher conserved amino acid sequences were not found except for the N-terminal of GSE, although no domain or motif specific to these amino acids was predicted in the database. Thus, this analysis showed a conserved role of GSE among various vertebrate species, including mice and humans.

**Figure 1:**
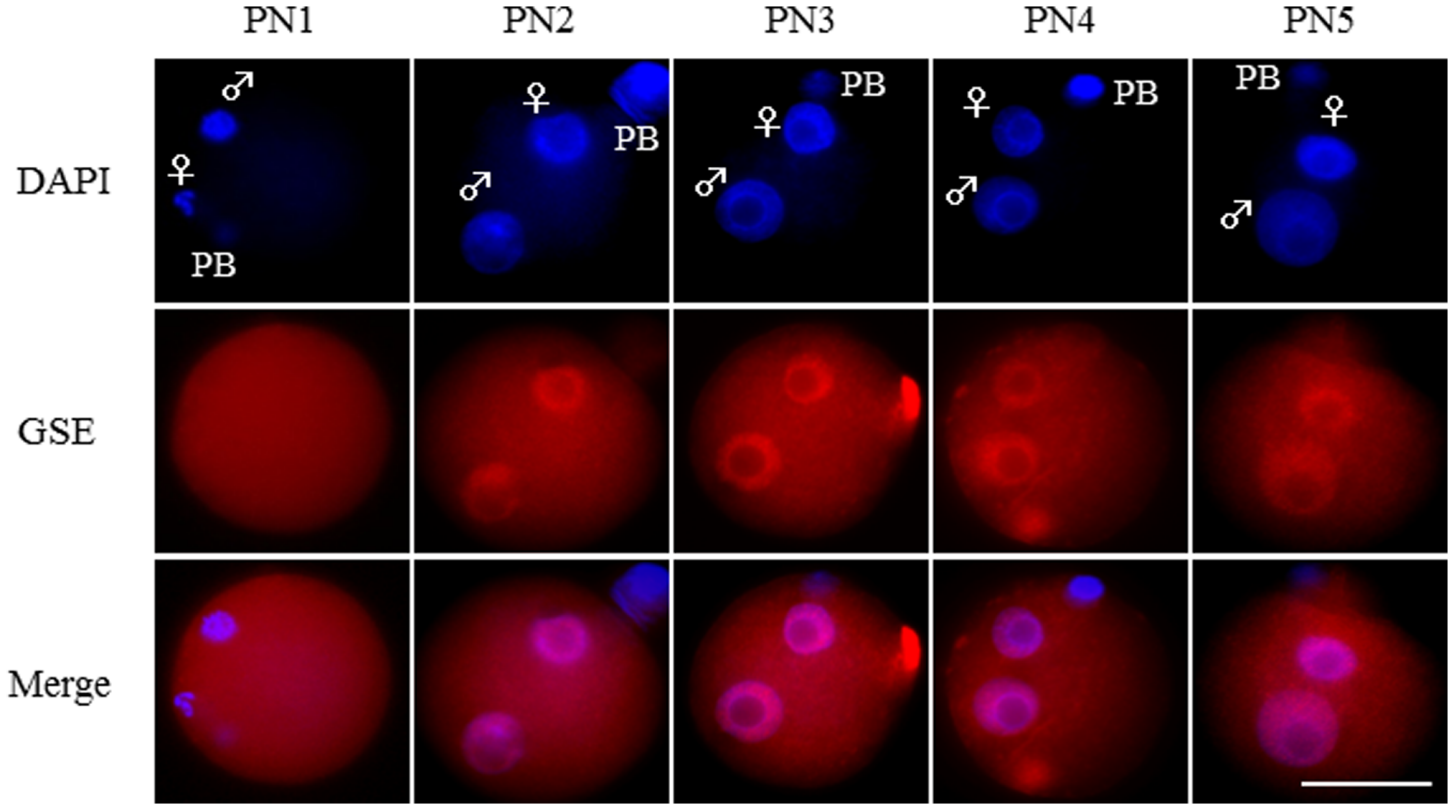
Localization of GSE in fertilized mouse oocytes. The dynamic appearance of the pronuclear localization of GSE is illustrated during mouse zygotic development. Shown are representative images of zygotes stained with DAPI (blue) and with anti-GSE antibody immunostaining (red). Key: ♀, female pronucleus; ♂, male pronucleus; PB, polar body; scale bars = 50 µm. The numbers of zygotes analyzed for each stage were: PN1, 11; PN2, 11; PN3, 10; PN4, 10; and PN5, 10.

Next, we examined the expression profile of the GSE protein in preimplantation mouse embryos by western blot analysis. Consistent with previous immunocytochemical analysis [15], GSE protein was constitutively expressed in germinal vesicle (GV) stage and stage 2 meiosis (MII) oocytes and pre-implantation embryos until blastocyst stage, although the abundance of this protein was decreased at the blastocyst stage (Figure S1C). In our previous study, we also showed that the predominant localization of GSE in the nucleus was observed throughout preimplantation cleavage stages. To assess the pronuclear localization of GSE in mouse zygotes, we examined its subcellular localization during PN1–5 [8], [11]. In zygotes, the GSE protein showed a unique pronuclear localization profile, in which its signal was clearly observed from PN2 onward (Figure 1).

**Figure 2:**
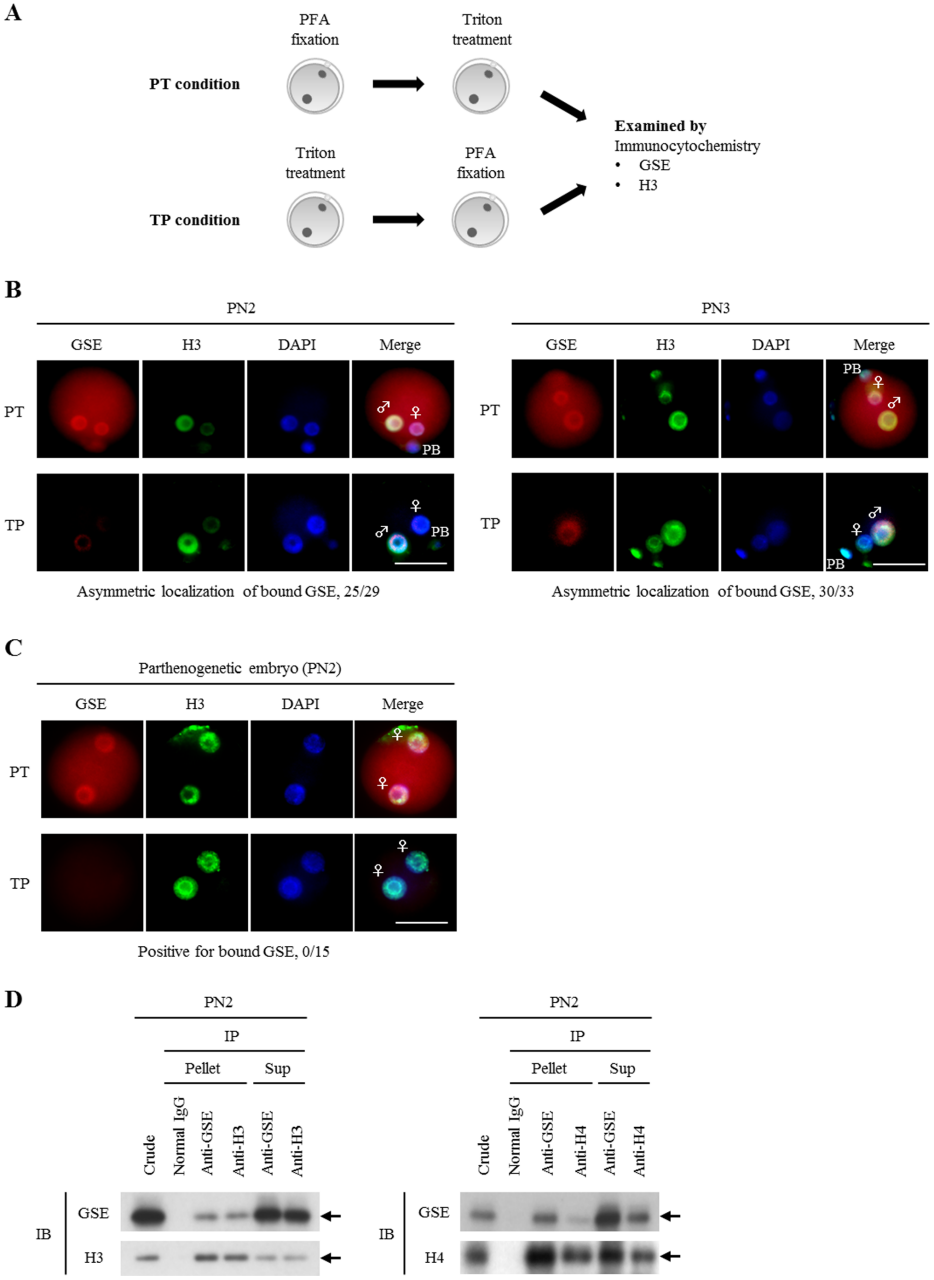
Preferential binding of GSE to the paternal chromatin. (A) Brief scheme of two pretreatment procedures before immunocytochemical staining: pretreatment with Triton X-100 before PFA fixation (TP condition) versus conventional PFA fixation (PT condition). (B) Immunocytochemical analysis of GSE (red) in zygotes at PN2 and PN3 after pretreatment under the PT or TP conditions. Histone H3 is shown in green as a control for chromatin-bound factors under the TP condition. Numbers of zygotes analyzed for each group were: PN2 (PT condition), 20; PN2 (TP condition), 25; PN3 (PT condition), 20; PN3 (TP condition), 30. DNA was stained with DAPI (blue). Key: ♀, female pronucleus; ♂, male pronucleus; PB, polar body; scale bars = 50 µm. (C) Immunocytochemical analysis of GSE in parthenogenetic embryos after pretreatment under the PT or TP conditions. H3 is shown in green as a control for chromatin-bound factor under the TP condition. Numbers of zygotes analyzed for each group: PT condition, 15; TP condition, 15. DNA was stained with DAPI (blue). Key: ♀, female pronucleus; PB, polar body; scale bars = 50 µm. (D) Immunoprecipitation with an anti-GSE antibody followed by immunoblotting using anti-H3 or -H4 antibodies in zygotes at PN2 and PN3. Normal rabbit IgG was used as a negative control. Arrows indicate each respective band. Three independent experiments were performed.

We then examined whether GSE was associated with pronuclear chromatin architecture during zygote development. Fertilized oocytes were subjected to immunocytochemical analysis using the pre-extraction method [13], [27], involving pretreatment with Triton X-100 before PFA fixation (TP condition), in contrast with the conventional PFA fixation (PT condition) (Figure 2A). This showed that the localization pattern of GSE in zygotes was clearly different under the two conditions. Under the TP condition, a faint signal was visible in only the paternal pronucleus at PN2 (Figure 2B left) and the signal became stronger in the paternal pronucleus at PN3 (Figure 2B right), indicating that chromatin-bound GSE was present in the paternal pronucleus and that GSE in the maternal pronucleus had been eluted by the detergent (Figure 2B). Interestingly, signals of GSE and PGC7/Dppa3/Stella proteins, the latter which has already been shown to dominantly bind to the maternal genome [13], were oppositely observed in the paternal and maternal pronuclei at PN2 under the condition of TP, respectively (Figure 2B and S2) although our observations were not performed on single zygotes. Furthermore, GSE was not detected in two maternally derived pronuclei in parthenogenetic embryos at PN2 under the TP condition, although GSE was detectable in them under the conventional PT condition (Figure 2C). These results indicated that GSE was preferentially bound to the paternal rather than the maternal chromatin. The observation that GSE was able to associate with chromatin architecture in zygotes was also confirmed in reciprocal coimmunoprecipitation experiments using anti-GSE and anti-H3 or -H4 antibodies (Figure 2D). We also confirmed by *in vitro* assay that recombinant GSE was directly bound to the N-terminal (1–100 amino acids) of unmodified H3 or H4 peptides (Figure S3). These results showed that GSE was partially associated with H3 or H4 in the pronuclei at PN2.

**Figure 3:**
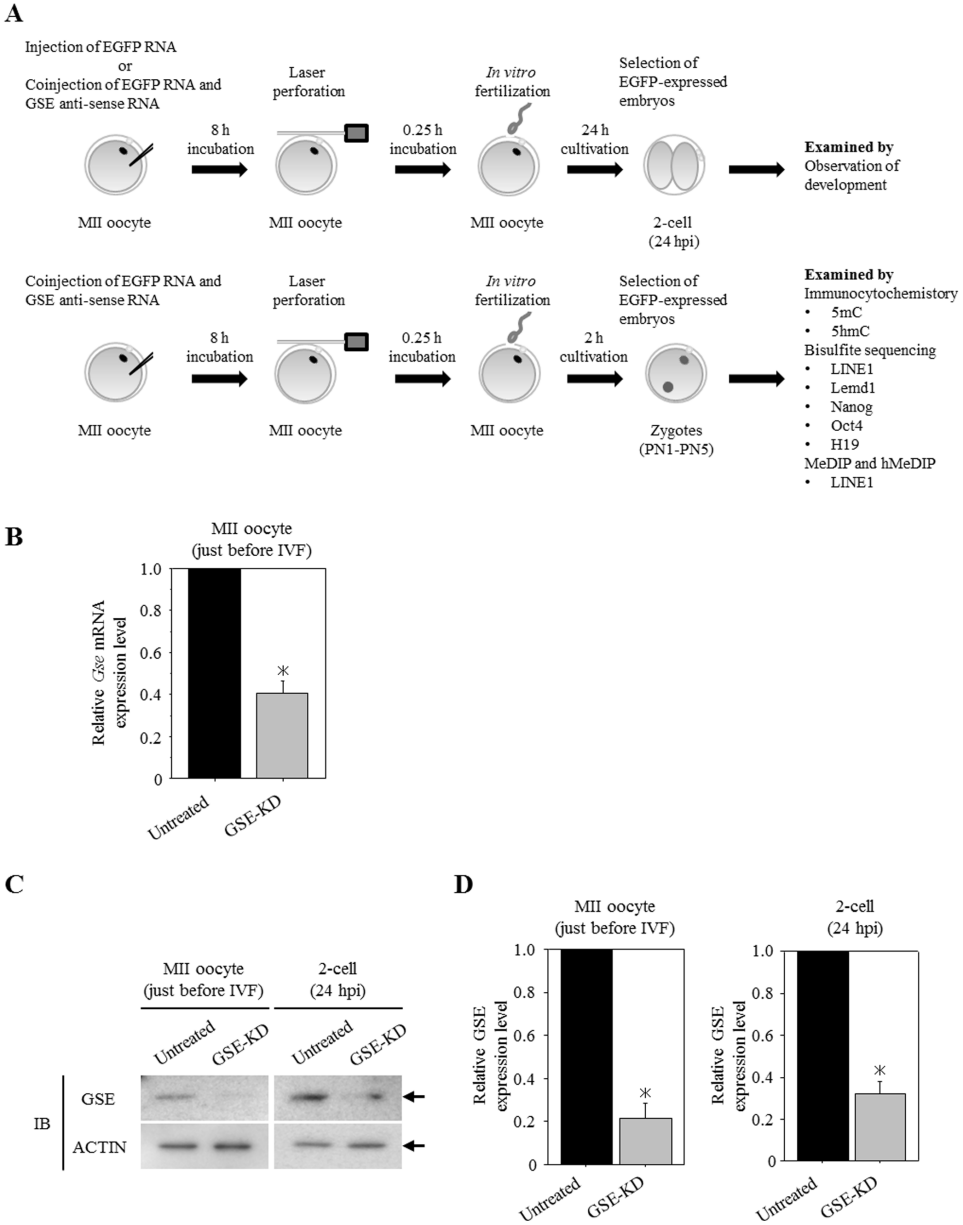
Maternal GSE-knockdown (GSE-KD) zygotes obtained from antisense RNA injection and in vitro fertilization (IVF). (A) Scheme of the experimental procedures. In immunocytochemistory, bisulphite sequencing, and MeDIP and hMeDIP (methylated and hydroxymethylated DNA immunoprecipitation) analyses, EGFP RNA-uninjected MII oocytes, incubated under similar conditions, were used as untreated controls. (B) Knockdown of GSE expression by an antisense RNA was confirmed by quantitative RT-PCR analysis. The relative ratios were obtained by dividing the expression level of the *Gse* gene by the expression level of the *G3PDH* gene. More than 90 oocytes from three independent experiments were analyzed. Shown are statistically significant differences between untreated and GSE-KD oocytes (*p<0.05). Bars represent the standard error of the mean. (C) Knockdown of GSE protein expression was confirmed by immunoblot analysis of MII oocytes just before IVF or in 2-cell embryos. Ninety oocytes from three independent experiments were analyzed. Actin was used as a loading control. (D) Densitometric quantification of the immunoblot bands of Figure 3C showing statistically significant differences between untreated and GSE-KD oocytes or embryos (*p<0.05). Bars represent the standard error of the mean.

Our finding that GSE was preferentially bound to the paternal chromatin architecture during zygote development prompted us to investigate the involvement of GSE in paternal genome demethylation during epigenetic reprogramming after fertilization. To clarify the role of GSE during early embryo development, GSE-knockdown (GSE-KD) zygotes were generated by cytoplasmic injection of GSE antisense RNA together with EGFP RNA as a marker of successful injection (Figure 3A and S4). In MII oocytes, 60% and 79% losses of GSE mRNA and proteins in the coinjected EGFP-expressing oocytes were detected by quantitative RT-PCR and immunoblot analysis, respectively (Figure 3B, 3C left and 3D left). At the 2-cell stage, 68% loss of GSE proteins in the EGFP-positive embryos was detected by immunoblot analysis (Figure 3C right and 3D right). Most of the EGFP-positive oocytes were fertilized and developed to blastocysts normally, as well as the control embryos injected with EGFP RNA alone and showing EGFP expression (Table 1). This result suggested that the loss of GSE had no evident effect on at least the first and second cell cycles of preimplantation mouse embryos.

**Table 1:** Effect of GSE knockdown on the development of early mouse embryos.

Injected RNAs	No. (%) of oocytes		No. (%) of fertilized oocytes	No. (%) of 2-cell embryos expressing EGFP	No. (%) of embryos developing to:			
	Injected	Survived			4-cell (48 hpi)	8-cell (60 hpi)	Morulae (72 hpi)	Blastocysts (96 hpi)
Untreated	–	–	51 (64)*	–	47 (92)	43 (84)	34 (67)	23 (45)
EGFP RNA	170	60 (35)	36 (60)	34 (57)	29 (85)	26 (76)	18 (53)	13 (38)
EGFP RNA+GSE antisense RNA (GSE-KD)	162	61 (38)	36 (59)	33 (54)	27 (82)	23 (70)	18 (55)	13 (39)

* In the untreated group, M II oocytes were cultured for 8.25 h and fertilized *in vitro*.

Key: EGFP, enhanced green fluorescent protein; hpi, hours postinsemination; GSE-KD, GSE knockdown.

**Figure 4:**
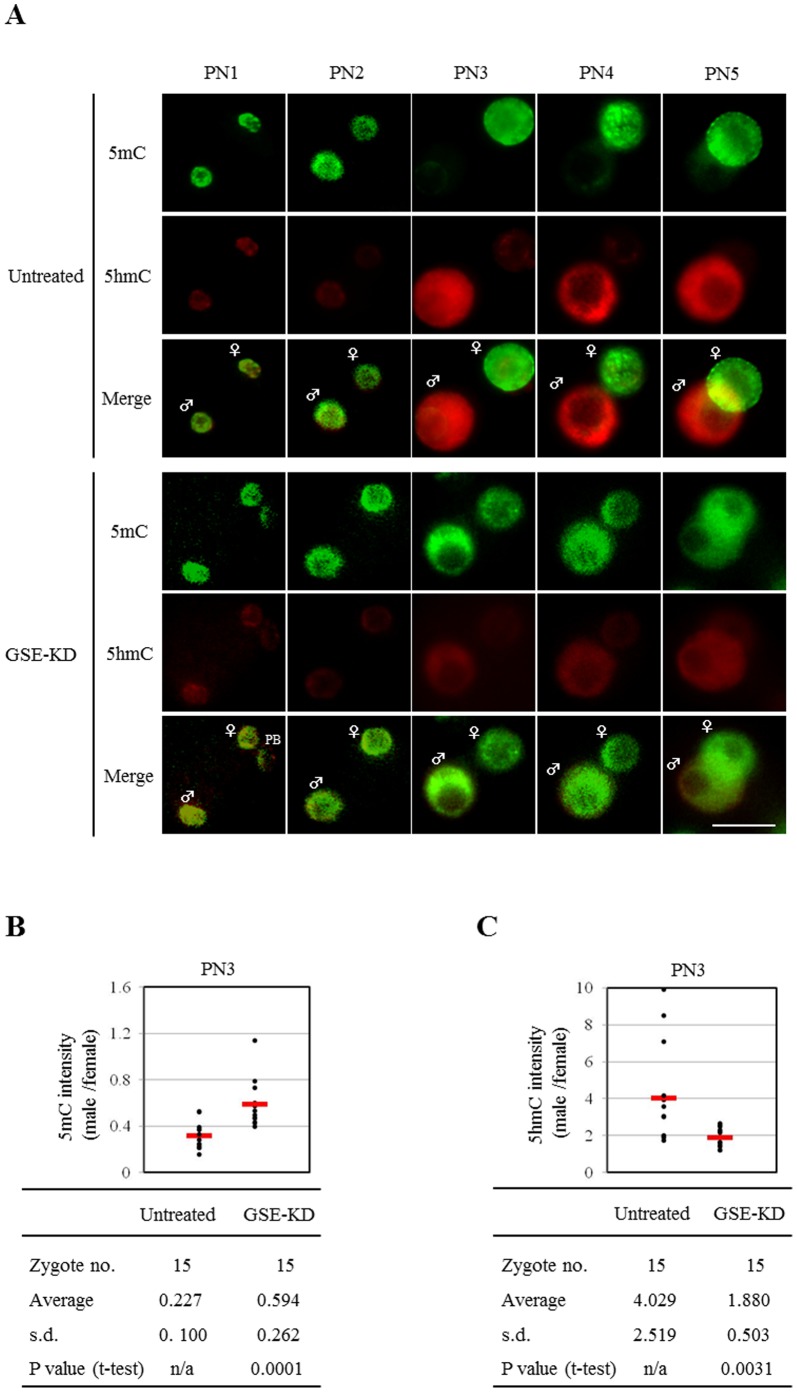
Effect of GSE knockdown on paternal DNA demethylation during zygote development. (A) Immunofluorescent images of 5****mC (green) and 5****hmC (red) in untreated and GSE-KD zygotes. Green fluorescence images of anti-5****mC are shown in false-color. The respective numbers of untreated and GSE-KD zygotes analyzed for each stage were: PN1, 15 and 10; PN2, 15 and 10; PN3, 15 and 15; PN4, 10 and 10; PN5, 10 and 10, respectively. Key: ♀, female pronucleus; ♂, male pronucleus; PB, polar body; scale bars = 25 µm. (B) Quantification of the ratio (male to female) of 5****mC intensity in untreated and GSE-KD zygotes at PN3. Each dot plot represents a single zygote. Red bars represent the mean ratio of each zygote. (C) Quantification of the ratio (male to female) of 5****hmC intensity in untreated and GSE-KD zygotes at PN3. Each dot plot represents a zygote. Red bars represent the mean ratio for each zygote.

To uncover the function of GSE in the paternal genome demethylation during zygote development, GSE-KD zygotes were subjected to immunocytochemical analysis for 5****mC and 5****hmC along with untreated control zygotes. In untreated zygotes, the loss of 5****mC and the appearance of 5****hmC clearly occurred in the paternal pronucleus from PN2 to PN3 but not in the maternal pronucleus at PN1–5, consistent with previous reports [1], [8], [11]. However, compared with untreated zygotes, the dynamic loss of 5****mC signals and the coincident accumulation of 5****hmC signals in the paternal pronucleus could not be detected in GSE-KD zygotes at any pronuclear stages (Figures 4A and S5). We could also not observe any obvious effects of GSE knockdown on 5****mC and 5****hmC signals in the maternal pronucleus. Furthermore, the ratio (paternal to maternal) of 5****mC intensity in pronuclei was significantly higher in the GSE-KD zygotes than in untreated controls (Figure 4B), whereas the level of 5****hmC intensity in the paternal pronucleus relative to the maternal one was significantly decreased by knockdown of GSE expression (Figure 4C). Thus, our findings suggest that maternal GSE is involved in the molecular mechanisms of the conversion of 5****mC to 5****hmC during zygote development.

**Figure 5:**
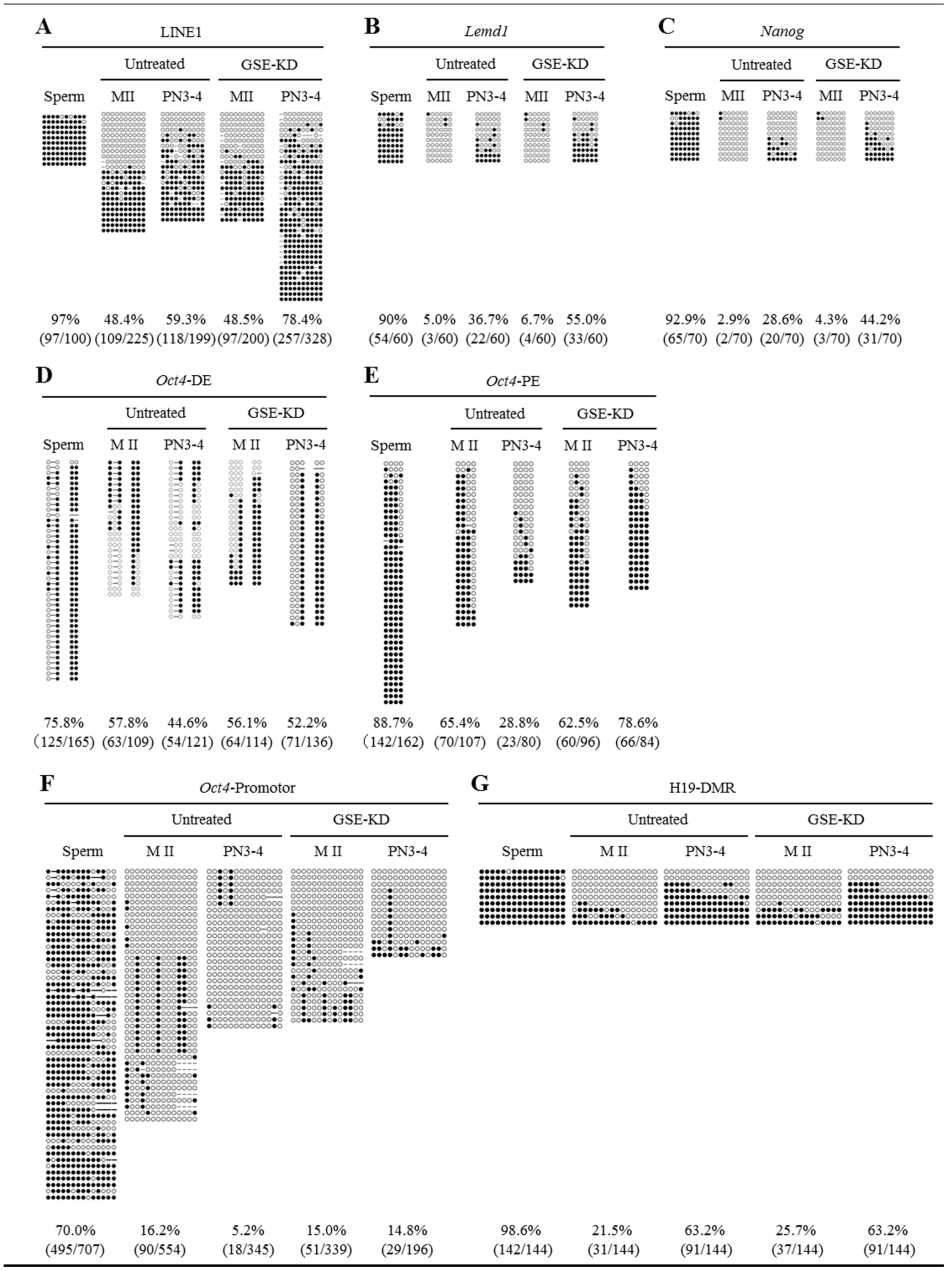
Bisulphite sequencing analysis of LINE1, *Lemd1*, *Nanog*, *Oct4* and H19*-*DMR in GSE-KD oocytes and zygotes. (A) Bisulphite sequencing of CpG sites within LINE1 genomic regions in spermatozoa and in untreated and GSE-KD oocytes and zygotes at PN3–4. (B) Bisulphite sequencing of CpG sites within *Lemd1* genomic regions in spermatozoa and in untreated and GSE-KD oocytes and zygotes at PN3–4. (C) Bisulphite sequencing of CpG sites within *Nanog* genomic regions in spermatozoa and in untreated and GSE-KD oocytes and zygotes at PN3–4. The methylation profile of the CpG sites in detail is indicated in Figure S8. (D, E, F) Bisulphite sequencing of CpG sites within the upstream distal enhancer (DE) (D), proximal enhancer (PE) (E) and promoter regions (F) of the *Oct4* gene in spermatozoa and untreated and GSE-KD oocytes and zygotes at PN3–4. (G) Bisulphite sequencing of CpG sites within the H19-DMR in sperm and untreated and GSE-KD oocytes and zygotes at PN3–4.

To obtain evidence that GSE knockdown indeed affects DNA demethylation, we evaluated DNA methylation status in specific sequences by bisulphite sequencing. The CpG sites of LINE1 transposable elements, the testis-specific gene *Lemd1*, and the pluripotent related genes *Nanog* and *Oct4* are demethylated by active DNA demethylation in zygotes at the pronuclear stage [9], [26], [27]. Therefore, we first performed bisulphite sequencing analysis of the CpG sites within LINE1, *Lemd1*, *Nanog* and the upstream regulatory region of *Oct4*, including the distal enhancer (DE), proximal enhancer (PE) and promoter regions [25], in untreated and GSE-KD zygotes at mid-pronuclear stages (PN3–4) (Figure 5). Importantly, there were no critical differences in the DNA methylation status of the examined genes between untreated and GSE-KD oocytes, indicating that maternal GSE was not involved in maintaining the DNA methylation status in MII oocytes. For a comparison, we also examined DNA methylation status in mouse spermatozoa and MII oocytes. The DNA methylation levels in CpG sites of LINE1, *Lemd1*, *Nanog*, and the DE, PE and promoter regions of *Oct4* in untreated zygotes at PN3–4 were 59.3%, 36.7%, 28.6%, 44.6%, 28.8% and 5.2%, respectively, markedly lower than the mean methylation levels calculated for spermatozoa and untreated MII oocytes (72.8%, 47.5%, 47.9%, 66.8%, 77.1% and 43.1%, respectively). Thus, DNA demethylation had occurred at mid-pronuclear stages (PN3–4), consistent with previous findings [9], [26], [27]. However, in GSE-KD zygotes at PN3–4, DNA demethylation levels in the CpG sites of LINE1, *Lemd1*, *Nanog*, and the DE, PE and promoter regions of *Oct4* were 78.4%, 55.0%, 44.2%, 52.2%, 78.6% and 14.8%, respectively, almost corresponding to the mean levels observed for spermatozoa and GSE-KD oocytes (72.8%, 48.4%, 48.6%, 66.0%, 75.6% and 42.5%, respectively), although DNA demethylation in the promoter regions of *Oct4* was hampered but not completely impeded by knockdown of GSE. Thus, knockdown of GSE resulted in an impairment of DNA demethylation in zygotes, but the significance of GSE in the genomic region-dependent DNA demethylation mechanism remains to be elucidated.

We next examined whether knockdown of GSE would impair DNA methylation status in the CpG sites of the paternally imprinted, differentially methylated H19 region (H19-DMR). In agreement with previous studies in which H19-DMR was demethylated in PGCs but not it zygotes [28], [29], bisulphite sequencing analysis showed that H19-DMR was methylated at a level of 63.2% in untreated zygotes at PN3–4, close to the mean methylation level calculated for spermatozoa and untreated oocytes (60.1%; Figure 5). GSE-KD zygotes at PN3–4 had a mean methylation level of 63.2%, very similar to the mean level observed for spermatozoa and GSE-KD zygotes (62.2%). Thus, these results indicate that the DNA methylation status in LINE1, *Lemd1*, *Nanog*, and the upstream regulatory regions of *Oct4* was specifically affected by GSE knockdown.

**Figure 6:**
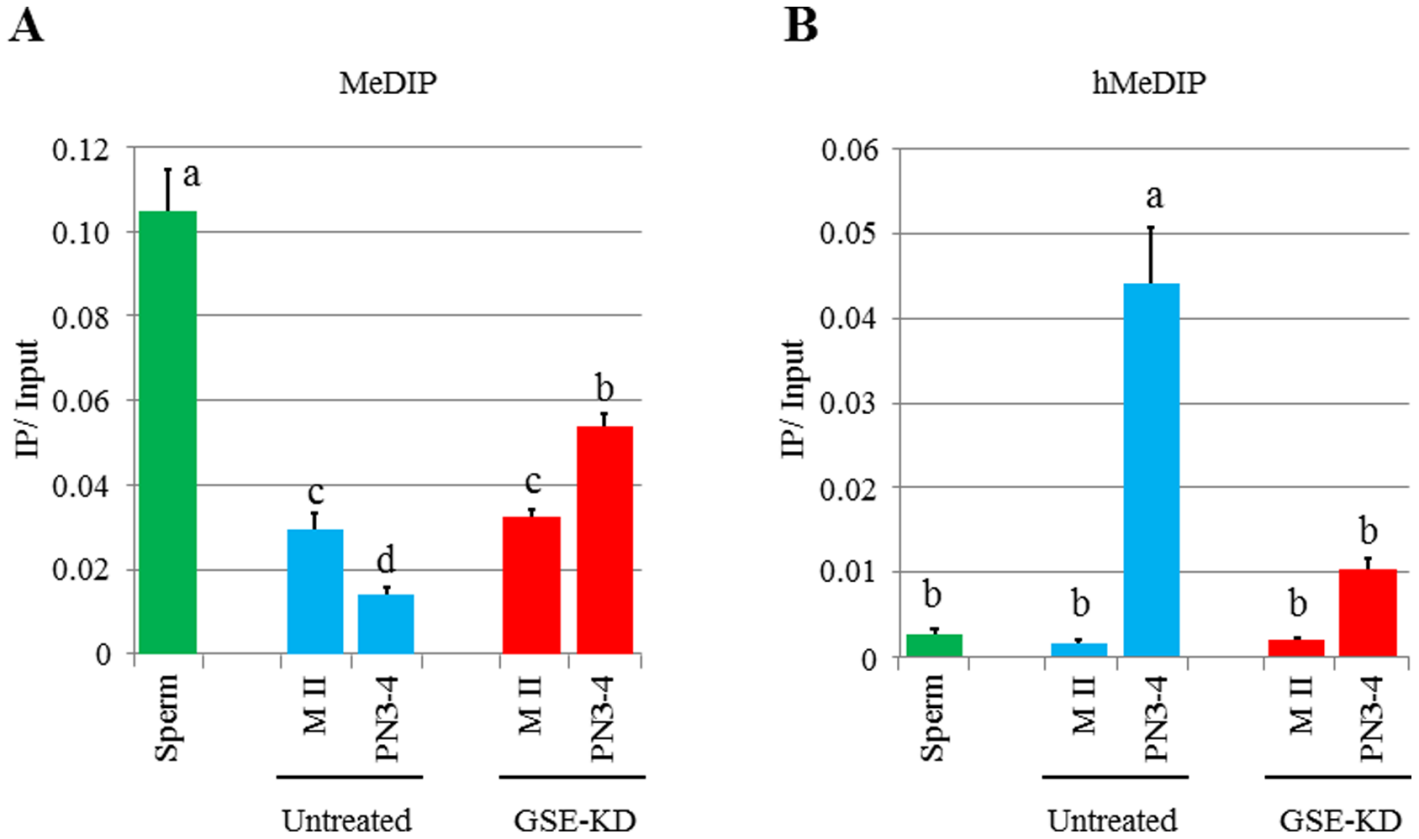
DNA immunoprecipitation on LINE1 genomic regions in GSE-KD oocytes and zygotes. (A) Quantitative PCR analysis of LINE1 genomic regions on immunoprecipitation using anti-5****mC antibody (for MeDIP). 30 PN3–4 embryos and 30 MII oocytes were collected to extract genomic DNA. Different letters indicate statistical significance (*p*<0.05). Bars represent the standard error of the mean. (B) Quantitative PCR analysis of LINE1 genomic regions on immunoprecipitation using anti-5****hmC antibody (for hMeDIP). 30 PN3–4 embryos and 30 MII oocytes were collected to extract genomic DNA. Different letters indicate statistical significance (*p*<0.05). Bars represent the standard error of the mean.

Bisulphite sequencing analysis cannot distinguish between 5****mC and****5hmC [30], [31]. Therefore, to examine whether GSE is indeed involved in the conversion of 5****mC to 5****hmC, we subjected the genomic DNA from spermatozoa, untreated MII oocytes and zygotes at PN3–4, and GSE-KD MII oocytes and zygotes at PN3–4 to methylated and hydroxymethylated DNA immunoprecipitation (MeDIP and hMeDIP) at LINE1. The 5****mC level in untreated zygotes at PN3–4 was significantly lower than those of both spermatozoa and untreated MII oocytes, whereas the 5****hmC level in untreated zygotes at PN3–4 was significantly lower than those of both spermatozoa and untreated MII oocytes (Figure 6A and B). These are consistent with a previous observation by MeDIP and hMeDIP analyses that the conversion of 5****mC to 5****hmC occurs after fertilization [9]. In this experiment, we also observed that knockdown of GSE caused a significant increase of 5****mC level and concurrently a significant reduction of 5****hmC level in zygotes at PN3–4, while the 5****mC and 5****hmC levels in untreated MII oocytes were not different from those in GSE-KD MII oocytes (Figure 6A and B). These results indicate that the conversion of 5****mC to 5****hmC at LINE1 is impeded by GSE knockdown.

These data collectively indicate that maternal GSE binds preferentially to the paternal chromatin before or just at the beginning of active demethylation at PN2–3 [32] and suggest that maternal GSE plays a role in DNA demethylation during zygote development independently of the first round of DNA replication.

## Discussion

We demonstrated here that although GSE was present in both pronuclei, the protein was associated tightly and predominantly with the paternal chromatin architecture from PN2 onward, at which point active DNA demethylation commenced. GSE proteins, whose amino acid sequences lack any known enzymatic domains, are highly conserved among mammalian species. GSE is a relatively small protein (∼27 kDa), compared with a complete histone octamer (approximately 100 kDa). Interestingly, the chromatin-bound PGC7/Dppa3/Stella is also a small protein (∼17 kDa) and is predominantly confined to the maternal pronucleus during zygote development, resulting in a change in chromatin configuration [12], [13]. These above findings lead us to propose that GSE and PGC7/Dppa3/Stella might act in a competitive fashion to block one another’s association with chromatin. Indeed, we observed that GSE was not involved in DNA demethylation of the paternally methylated H19 locus, which is protected by the binding of PGC7/Dppa3/Stella to H3K9me2-marked chromatin in the paternal pronucleus [12], [13]. Although H3K9me2 allows tight PGC7/Dppa3/Stella association with chromatin, further studies will need to address how GSE could be tightly attached to chromatin. It also remains to be clarified why GSE is more strongly associated with the paternal pronucleus. Protamines that help pack the paternal sperm DNA are replaced by the H3.3 histone variant soon after fertilization [33], [34]. As preferential localization of H3.3 in the paternal pronucleus occurs at PN2 [26], it seems important to investigate whether GSE could be associated with this process.

We also observed that GSE knockdown impaired active DNA demethylation as assessed by five independent assays (5****mC staining, 5****hmC staining, bisulphite sequencing, MeDIP and hMeDIP). How GSE mediates active DNA demethylation in zygotes has yet to be determined. However, as the molecular mechanism of DNA demethylation in the paternal pronucleus involves the conversion of 5****mC to 5****hmC catalyzed by the maternal Tet3 enzyme [9]–[11], we speculate that GSE might be involved in the active DNA demethylation process associated with Tet3. Indeed, GSE-knockdown led to the impairment of DNA demethylation status in the CpG sites within LINE1, *Lemd1* and *Nanog*, in which DNA demethylation status is also shown to be impeded in Tet3-null zygotes [9]. Furthermore, in MeDIP and hMeDIP analyses using the same primers as reported by Gu *et al*.(2011) [9], we also observed the impairment of the conversion of 5****mC to 5****hmC at LINE1 in GSE-KD zygotes as well as in Tet3-null zygotes. Interestingly, we found here that the pronuclear localization of GSE occurs at PN2, which seems to be consistent with the observation of the concentration of Tet3 protein in the male pronucleus of PN3 zygotes [9]. Further studies are needed to confirm our hypothesis. In this study, we investigated the possible function of GSE in GSE-KD zygotes achieved by treating them with antisense RNA before fertilization. Knockout model mice are under construction to test whether chromatin-bound GSE is involved in the active DNA demethylation associated with Tet3.

In conclusion, understanding the function of GSE in gametic DNA demethylation in cooperation with Tet3 and PGC7/Dppa3/Stella during zygote development will help elucidate the molecular mechanisms of epigenetic reprogramming of the male and female germ cells to generate a totipotent zygote.

## Supporting Information

Figure S1 **Alignment of GSE orthologs and expression of GSE in oocytes and preimplantation embryos.** (A) Amino acid sequence alignment of mouse, rat, human, pig and bovine GSE. To maximize the quality of the alignment, gaps (shown as dashes) were introduced in the sequences. The multiple sequence alignment obtained by ClustalW2 program (http://www.ebi.ac.uk/Tools/clustalw2/index.html) is shown. Completely conserved residues are shaded in black; residues that are conserved in three and four of the sequences are shaded in gray. The amino acid sequences of the GSE orthologs have the following NCBI accession numbers: mouse GSE, NP_083574; rat GSE, NP_001157034; human GSE, NP_001157033; pig GSE, XP_003481269; bovine GSE, XP_003582826. (B) Amino acid sequence alignment of mouse, *Xenopus*, *zebrafish* and *Ciona intestinalis* GSE. To maximize the quality of the alignment, gaps (shown as dashes) were introduced in the sequences. The multiple sequence alignment obtained by ClustalW2 program is shown. Completely conserved residues are shaded in black; residues that are conserved in three and four of the sequences are shaded in gray. The amino acid sequences of the GSE orthologs have the following NCBI accession numbers: *Xenopus* GSE, XP_002932431; *zebrafish* GSE, XP_002664166; *Ciona intestinalis* GSE, XP_002126241. (C) Immunoblot analyses for protein expressions of GSE in mouse oocytes and early mouse embryos. Actin was used as a loading control in immunoblot analyses.
(TIF)

Figure S2 **Preferential binding of PGC7/Dppa3/Stella to the maternal chromatin.** Immunocytochemical analysis of PGC7/Dppa3/Stella (red) in zygotes at PN3 after pretreatment under the PT or TP conditions. Histone H3 is shown in green as a control for chromatin-bound factor under the TP condition. Numbers of zygotes analyzed for each group: PT condition, 15; TP condition, 15. DNA was stained with DAPI (blue). Key: ♀, female pronucleus; PB, polar body; scale bars = 50 µm.
(TIF)

Figure S3 ***In vitro*** **assay of the binding of His-tagged recombinant GSE to H3 and H4 peptides.** (A) Expression of His-tagged recombinant GSE protein in transfected HEK293 cells. His-tagged recombinant GSE protein purified from lysates of transfected HEK293 cells was detected by Coomassie Brilliant Blue (CBB) staining of the SDS gels. Arrow indicates each respective band. Asterisks indicate nonspecific bands. (B) Immunoblot analysis of expressed His-tagged recombinant GSE in transfected HEK293 cells. Actin was used as a loading control. (C) His-tagged recombinant GSE and the N-terminal (1–100 amino acids) of histone H3 peptide was used in immunoprecipitation analysis. Lysates of transfected cells were used for the immunoprecipitation with anti-His antibody, followed by immunoblotting using anti-H3 antibodies. Normal mouse IgG was used as a negative control. Arrows indicate each respective band. Three independent experiments were performed. (D) His-tagged recombinant GSE and the N-terminal (1–100 amino acids) of histone H4 peptides were used in immunoprecipitation analysis. Lysates of transfected cells were used for the immunoprecipitation with anti-His antibody, followed by immunoblotting using anti-H4 antibodies. Normal mouse and rabbit IgG were used as a negative control. Arrows indicate each respective band.
(TIF)

Figure S4 **Selection of EGFP-expressing oocytes or embryos.** (A) Laser perforation of RNAs-injected M II oocytes. Arrows represent the perforation of zona pellucida of each oocyte. Scale bars = 50 µm. (B) Selection of oocytes or embryos expressing *EGFP* gene. The RNAs coinjected oocytes or 2-cell embryos showing EGFP fluorescence were selected as GSE-KD cells. Scale bars = 100 µm.
(TIF)

Figure S5 **Representative images of 5 mC and 5 hmC staining in untreated and GSE-KD zygotes at PN3.** Shown are representative images of each zygote stained with anti-5****mC (green) and anti-5****hmC (red) antibodies. Green fluorescence images of anti-5****mC are shown in false-color. Key: ♀, female pronucleus; ♂, male pronucleus; PB, polar body; scale bars = 50 µm. Numbers of zygotes analyzed for each group: untreated, 14; GSE-KD, 14.
(TIF)

Table S1 **Information of antibodies.**
(XLS)

Table S2 **Information of primers.**
(XLS)
