# Supplementary figures and images for: GSE Is a Maternal Factor Involved in Active DNA Demethylation in Zygotes
Source: PLoS One. 2013 Apr 1;8(4):e60205. doi: 10.1371/journal.pone.0060205 (PMC3613368; doi:10.1371/journal.pone.0060205)

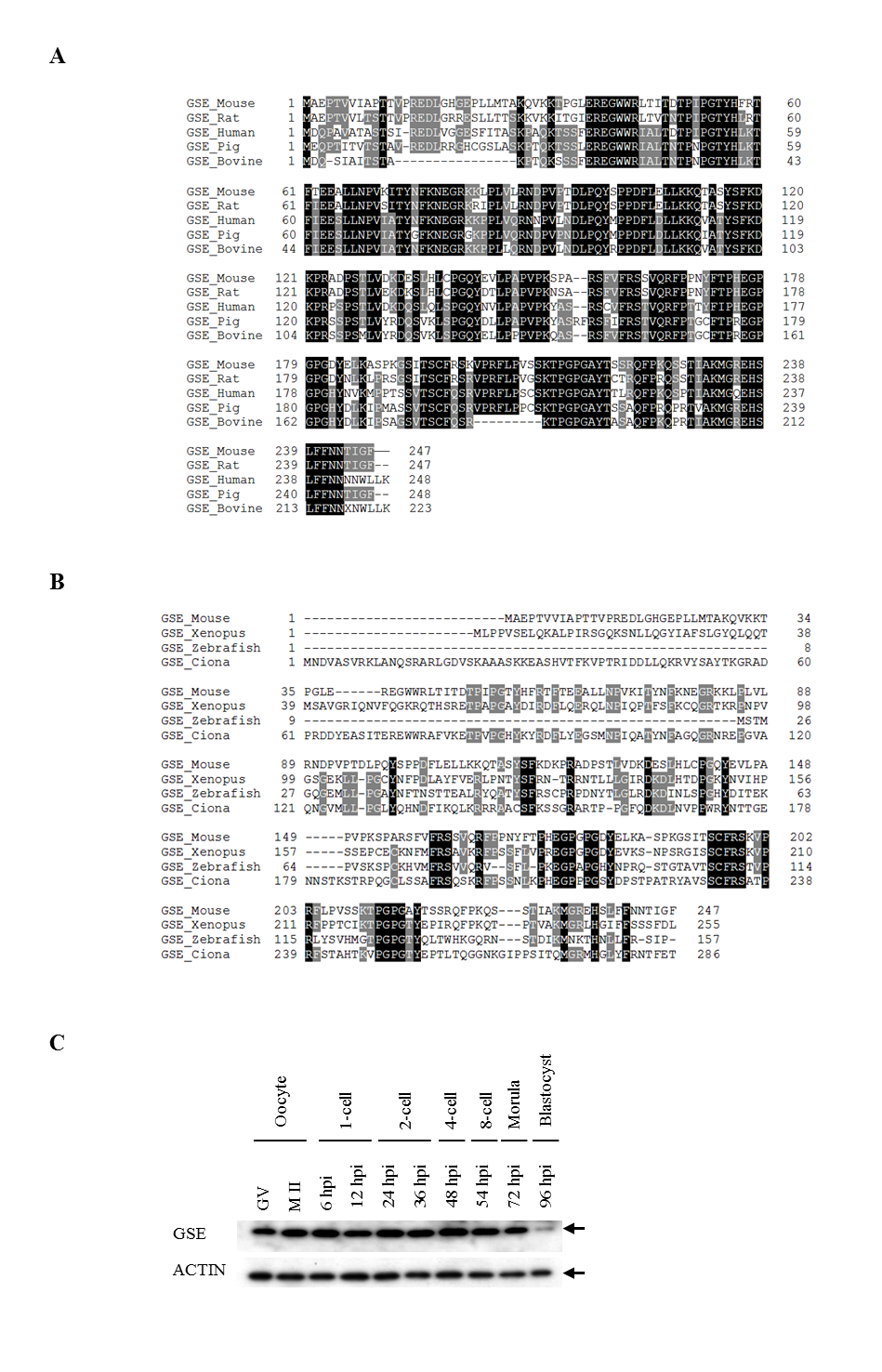

Supplement: Figure S1 — Alignment of GSE orthologs and expression of GSE in oocytes and preimplantation embryos. (A) Amino acid sequence alignment of mouse, rat, human, pig and bovine GSE. To maximize the quality of the alignment, gaps (shown as dashes) were introduced in the sequences. The multiple sequence alignment obtained by ClustalW2 program (http://www.ebi.ac.uk/Tools/clustalw2/index.html) is shown. Completely conserved residues are shaded in black; residues that are conserved in three and four of the sequences are shaded in gray. The amino acid sequences of the GSE orthologs have the following NCBI accession numbers: mouse GSE, NP_083574; rat GSE, NP_001157034; human GSE, NP_001157033; pig GSE, XP_003481269; bovine GSE, XP_003582826. (B) Amino acid sequence alignment of mouse, Xenopus, zebrafish and Ciona intestinalis GSE. To maximize the quality of the alignment, gaps (shown as dashes) were introduced in the sequences. The multiple sequence alignment obtained by ClustalW2 program is shown. Completely conserved residues are shaded in black; residues that are conserved in three and four of the sequences are shaded in gray. The amino acid sequences of the GSE orthologs have the following NCBI accession numbers: Xenopus GSE, XP_002932431; zebrafish GSE, XP_002664166; Ciona intestinalis GSE, XP_002126241. (C) Immunoblot analyses for protein expressions of GSE in mouse oocytes and early mouse embryos. Actin was used as a loading control in immunoblot analyses. (TIF) [file pone.0060205.s001.tif]

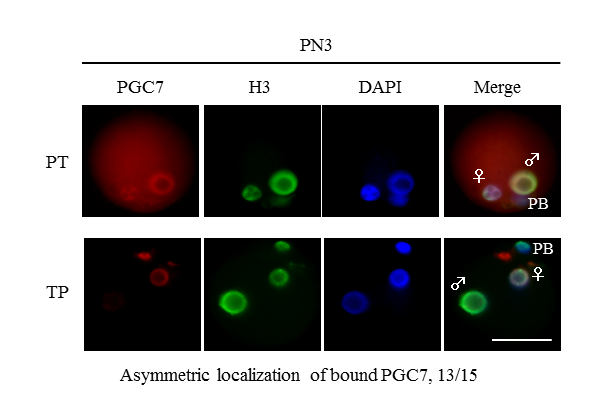

Supplement: Figure S2 — Preferential binding of PGC7/Dppa3/Stella to the maternal chromatin. Immunocytochemical analysis of PGC7/Dppa3/Stella (red) in zygotes at PN3 after pretreatment under the PT or TP conditions. Histone H3 is shown in green as a control for chromatin-bound factor under the TP condition. Numbers of zygotes analyzed for each group: PT condition, 15; TP condition, 15. DNA was stained with DAPI (blue). Key: ♀, female pronucleus; PB, polar body; scale bars = 50 µm. (TIF) [file pone.0060205.s002.tif]

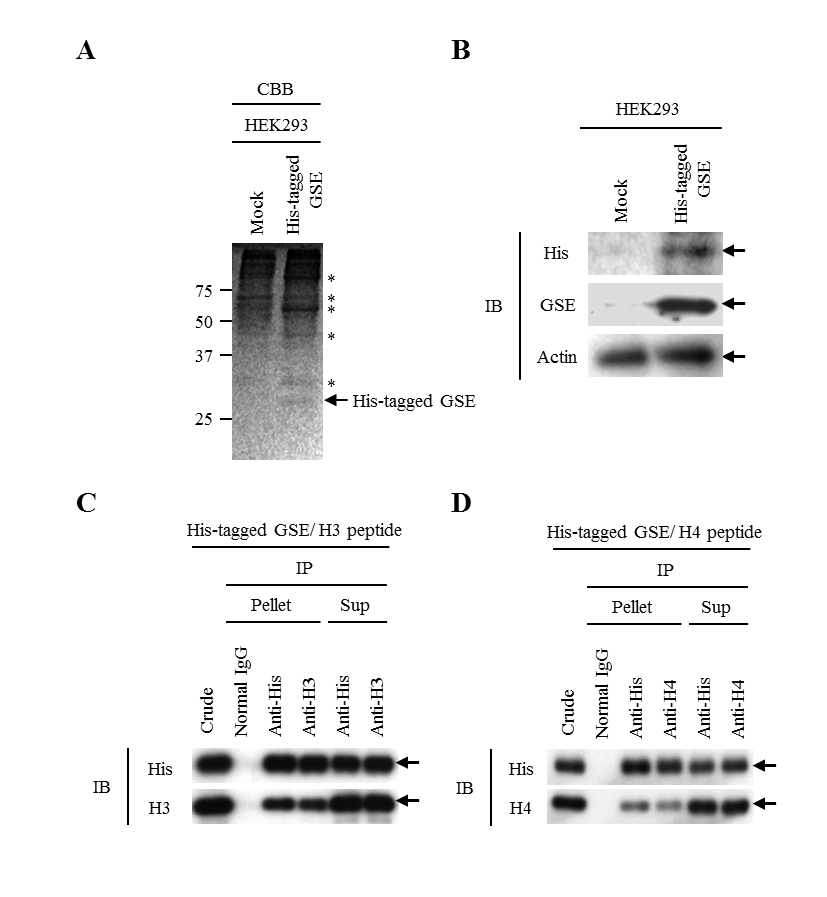

Supplement: Figure S3 — In vitro assay of the binding of His-tagged recombinant GSE to H3 and H4 peptides. (A) Expression of His-tagged recombinant GSE protein in transfected HEK293 cells. His-tagged recombinant GSE protein purified from lysates of transfected HEK293 cells was detected by Coomassie Brilliant Blue (CBB) staining of the SDS gels. Arrow indicates each respective band. Asterisks indicate nonspecific bands. (B) Immunoblot analysis of expressed His-tagged recombinant GSE in transfected HEK293 cells. Actin was used as a loading control. (C) His-tagged recombinant GSE and the N-terminal (1–100 amino acids) of histone H3 peptide was used in immunoprecipitation analysis. Lysates of transfected cells were used for the immunoprecipitation with anti-His antibody, followed by immunoblotting using anti-H3 antibodies. Normal mouse IgG was used as a negative control. Arrows indicate each respective band. Three independent experiments were performed. (D) His-tagged recombinant GSE and the N-terminal (1–100 amino acids) of histone H4 peptides were used in immunoprecipitation analysis. Lysates of transfected cells were used for the immunoprecipitation with anti-His antibody, followed by immunoblotting using anti-H4 antibodies. Normal mouse and rabbit IgG were used as a negative control. Arrows indicate each respective band. (TIF) [file pone.0060205.s003.tif]

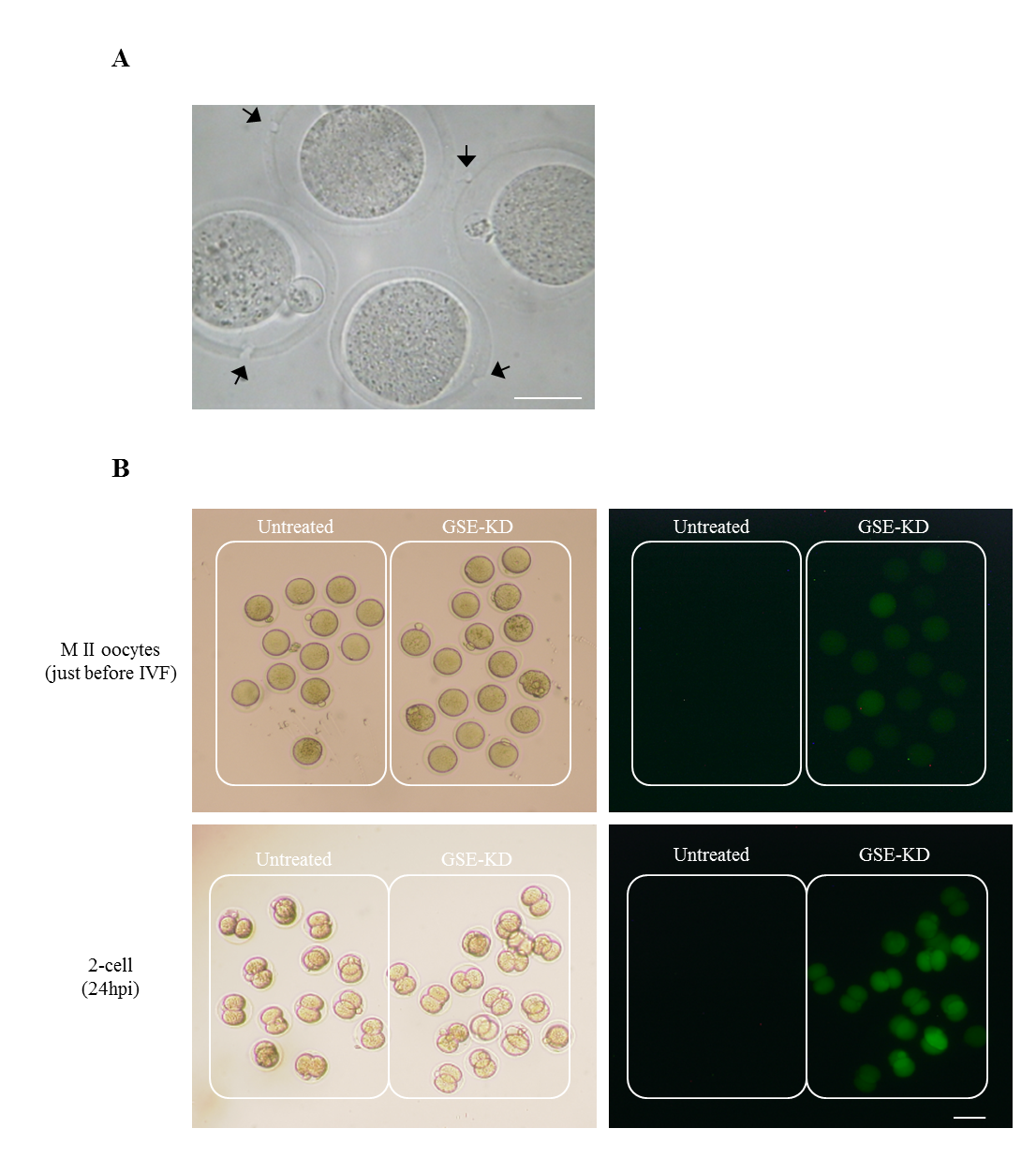

Supplement: Figure S4 — Selection of EGFP-expressing oocytes or embryos. (A) Laser perforation of RNAs-injected M II oocytes. Arrows represent the perforation of zona pellucida of each oocyte. Scale bars = 50 µm. (B) Selection of oocytes or embryos expressing EGFP gene. The RNAs coinjected oocytes or 2-cell embryos showing EGFP fluorescence were selected as GSE-KD cells. Scale bars = 100 µm. (TIF) [file pone.0060205.s004.tif]

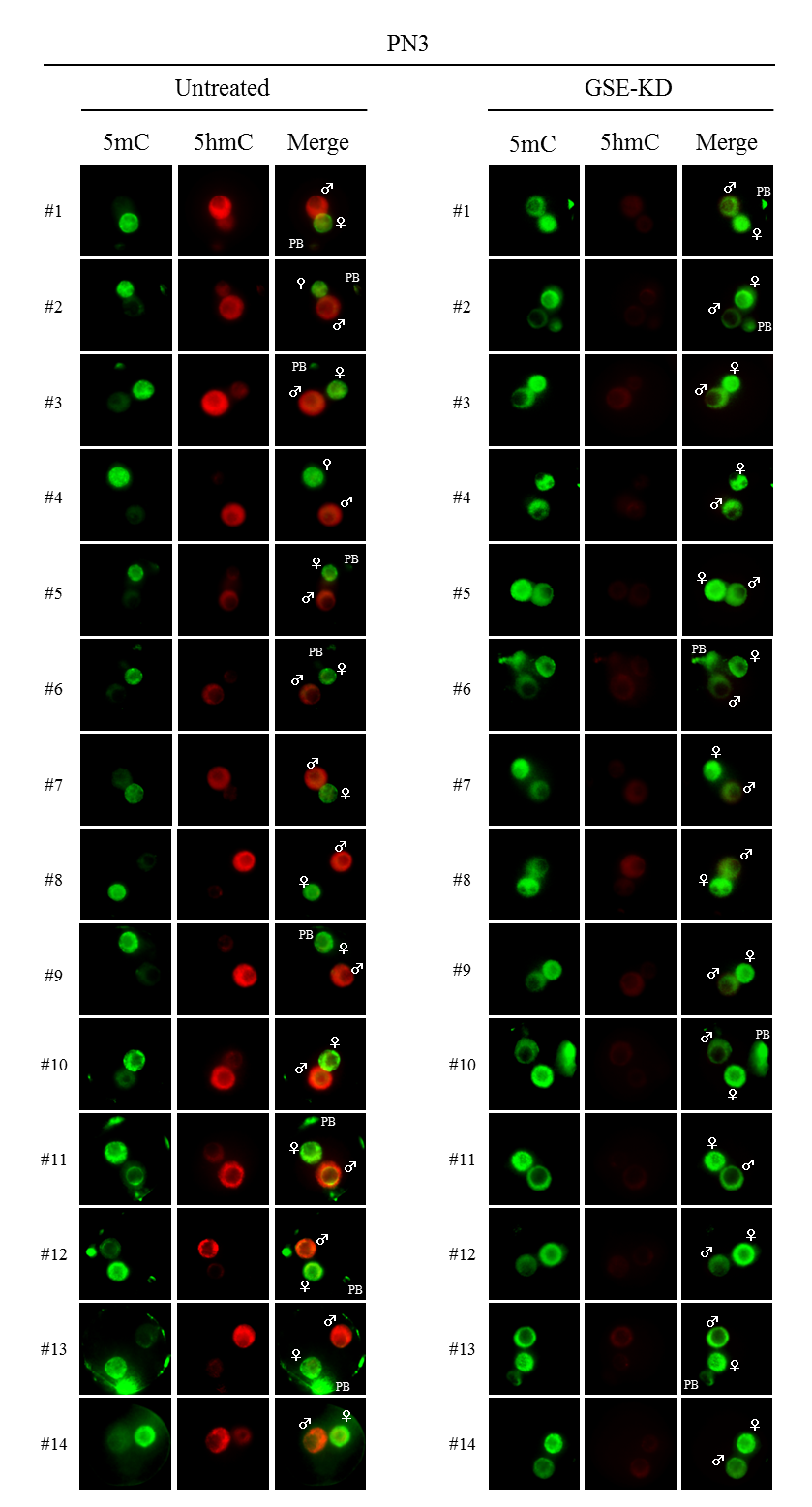

Supplement: Figure S5 — Representative images of 5 mC and 5 hmC staining in untreated and GSE-KD zygotes at PN3. Shown are representative images of each zygote stained with anti-5mC (green) and anti-5hmC (red) antibodies. Green fluorescence images of anti-5mC are shown in false-color. Key: ♀, female pronucleus; ♂, male pronucleus; PB, polar body; scale bars = 50 µm. Numbers of zygotes analyzed for each group: untreated, 14; GSE-KD, 14. (TIF) [file pone.0060205.s005.tif]
